# Supplementary material for: Improving water quality does not guarantee fish health: Effects of ammonia pollution on the behaviour of wild-caught pre-exposed fish
Source: PLoS One. 2021 Aug 9;16(8):e0243404. doi: 10.1371/journal.pone.0243404 (PMC8351958; doi:10.1371/journal.pone.0243404)
Supplement: S1 Table — (PDF) [file pone.0243404.s001.pdf]

S1 Table. Physiochemical water conditions throughout the experiment.

	TAN treatment	pH	Temperature (Cº)	NO_3^- (mg/L)	NO_2^- (mg/L)	NH_4^+ (mg/L)	NH_3 (mg/L)	Hardness
**Pre-exposed fish**	0 mg/L	8.30±0.23	20.67±0.52	5.08±0.65	0.00±0.00	0.024±0.046	0.002±0.004	14.75±1.50
	1 mg/L	8.29±0.23	20.93±0.34	4.83±0.91	0.00±0.00	1.429±0.624	0.122±0.092	16.50±4.80
	5 mg/L	8.30±0.22	20.97±0.37	5.00±0.92	0.00±0.00	5.004±1.174	0.400±0.205	14.25±2.50
	8 mg/L	8.25±0.19	20.87±0.38	4.92±1.12	0.00±0.00	8.074±1.248	0.679±0.237	15.00±2.82
**Non pre-exposed fish**	0 mg/L	8.34±0.15	21.41±0.77	4.73±0.90	0.00±0.00	0.014±0.039	0.001±0.003	15.06±3.76
	1 mg/L	8.38±0.17	21.72±0.72	4.69±0.83	0.00±0.00	1.366±0.293	0.156±0.057	15.50±4.30
	5 mg/L	8.41±0.19	21.54±0.44	4.69±0.83	0.00±0.00	4.995±0.486	0.581±0.213	14.25±2.50
	8 mg/L	8.33±0.15	21.73±0.47	4.69±0.83	0.00±0.00	8.066±0.726	0.708±0.208	15.05±3.76

Mean ± SD is presented for each TAN treatment (0, 1, 5, and 8 mg/L) and experimental group (pre-exposed and non pre-exposed fish). No differences in physiochemical parameters were found during the experiment between fish from the two groups and between the individual aquaria of each TAN treatment (GLM).
