# Supplementary material for: Improving water quality does not guarantee fish health: Effects of ammonia pollution on the behaviour of wild-caught pre-exposed fish
Source: PLoS One. 2021 Aug 9;16(8):e0243404. doi: 10.1371/journal.pone.0243404 (PMC8351958; doi:10.1371/journal.pone.0243404)
Supplement: S2 Table — (PDF) [file pone.0243404.s002.pdf]

S2 Table. Data collected from the swimming activity records of each specimen.

			Pre-exposed fish			Non pre-exposed fish		
TAN treatment	Specimen	Recording day	Swimming (s)	Not visible (s)	Resting (s)	Swimming (s)	Not visible (s)	Resting (s)
0 mg/L	1	1	2.08	9.25	6.27	12.47	5.13	0.00
		2	3.15	14.40	0.05	12.50	4.89	0.00
		3	6.32	10.37	0.51	13.26	3.08	1.26
		4	10.41	7.06	0.13	9.30	8.30	0.00
	2	1	13.44	4.16	0.00	9.39	7.44	0.38
		2	14.14	3.46	0.00	11.47	16.03	0.10
		3	12.35	5.25	0.00	14.43	3.42	0.36
		4	15.54	2.06	0.00	14.55	2.26	0.40
	3	1	14.55	1.23	1.42	13.04	3.42	1.14
		2	14.12	1.52	1.56	13.55	3.21	0.44
		3	14.46	0.57	2.17	14.33	2.38	0.49
		4	16.44	0.23	0.53	15.57	1.26	0.37
	4	1	14.24	2.36	0.59	5.16	12.40	0.04
		2	13.46	3.46	0.28	11.29	4.58	1.33
		3	13.27	3.38	0.55	15.39	1.56	0.25
		4	16.30	0.25	1.05	14.44	2.39	0.37
	5	1	3.56	13.12	0.52	8.13	9.31	0.16
		2	10.01	7.57	0.02	4.00	11.34	1.26
		3	9.27	8.21	0.12	4.17	13.38	0.05
		4	10.47	7.07	0.06	5.19	12.37	0.04
	6	1	3.23	13.19	1.18	10.35	4.51	2.34
		2	6.50	10.46	0.24	12.54	4.38	0.28
		3	11.08	6.02	0.50	15.07	2.21	0.32

		4	8.19	9.10	0.31	15.55	1.47	0.18
	7	1	12.13	5.30	0.17	13.56	3.31	0.33
		2	11.02	4.38	2.20	11.27	6.15	0.18
		3	9.59	6.07	1.54	16.06	1.22	0.32
		4	11.52	4.37	1.31	17.32	0.26	0.02
	8	1	16.39	1.21	0.00	14.13	1.10	2.37
		2	12.40	3.23	1.57	13.27	3.49	0.45
		3	12.04	5.21	0.35	16.47	1.06	0.07
		4	10.54	6.49	0.17	17.22	0.32	0.06
	9	1	11.43	1.16	5.01			
		2	14.47	0.19	2.54			
		3	13.39	2.49	1.32			
		4	16.20	0.17	1.23			
	10	1	7.11	8.41	2.08			
		2	9.24	7.37	0.59			
		3	6.45	11.00	0.15			
		4	12.24	4.55	0.41			
1 mg/L	1	1	8.18	7.46	1.56	13.58	4.02	0.00
		2	11.39	4.03	2.18	17.24	0.27	0.09
		3	9.14	8.46	0.32	16.19	1.36	0.05
		4	15.45	2.05	0.10	16.31	1.21	0.08
	2	1	3.17	1.54	12.49	10.38	6.54	0.29
		2	6.22	1.45	9.53	8.13	9.32	0.25
		3	3.03	14.53	0.04	15.34	2.13	0.13
		4	4.01	13.49	0.10	14.48	3.02	0.10
	3	1	13.21	3.22	1.17	16.04	1.28	0.28
		2	14.37	2.57	1.57	15.45	1.04	1.11

		3	14.32	2.28	0.59	16.57	0.50	0.13
		4	14.12	2.27	1.31	15.50	1.33	0.37
	4	1	8.01	17.49	0.10	4.43	11.33	1.44
		2	1.31	17.29	10.00	7.34	9.24	1.02
		3	6.15	9.08	2.37	12.28	5.26	0.06
		4	4.16	6.58	6.47	13.07	4.28	0.25
	5	1	12.37	3.25	1.58	9.12	8.23	0.25
		2	6.45	10.51	0.24	7.02	10.06	0.52
		3	6.07	11.53	0.00	7.19	10.13	0.28
		4	5.26	12.34	0.00	8.31	9.26	0.03
	6	1	2.24	15.16	0.20	8.56	6.34	2.30
		2	7.07	10.03	0.50	14.18	3.08	0.34
		3	6.44	0.00	0.04	15.20	1.19	1.21
		4	6.39	10.48	0.33	15.26	1.48	0.46
	7	1	13.56	1.29	2.35	10.01	7.21	0.38
		2	14.46	1.44	1.30	14.50	2.58	0.12
		3	12.38	4.23	0.59	15.29	2.21	0.10
		4	12.42	2.08	3.10	17.48	0.09	0.03
	8	1	16.49	1.05	0.06	1.43	16.17	0.00
		2	14.30	2.52	0.38	5.33	10.18	1.46
		3	16.44	0.27	0.49	11.08	5.42	1.10
		4	15.51	0.39	1.30	14.34	3.12	0.14
	9	1	4.17	13.37	0.06			
		2	6.25	4.03	7.32			
		3	7.34	10.24	0.02			
		4	14.37	2.42	0.41			
	10	1	5.45	11.58	0.17			

		2	10.02	7.54	0.04			
		3	11.20	6.38	0.02			
		4	8.45	9.09	0.06			
5 mg/L	1	1	12.03	5.50	0.07	8.17	9.38	0.21
		2	9.54	6.58	1.08	14.07	3.53	0.00
		3	2.19	15.41	0.00	11.02	6.57	0.01
		4	12.39	5.21	0.00	12.20	5.40	0.00
	2	1	10.42	6.11	1.07	10.05	7.47	0.08
		2	3.17	13.10	1.33	10.12	7.19	0.29
		3	13.05	3.10	1.45	17.17	0.40	0.03
		4	16.46	1.50	0.09	17.30	0.28	0.02
	3	1	5.48	8.06	4.06	15.24	1.34	1.02
		2	1.12	17.42	0.06	3.42	0.00	14.18
		3	14.30	2.58	0.33	16.50	0.47	0.23
		4	13.31	4.29	0.00	17.00	0.49	0.11
	4	1	17.39	0.11	0.10	14.53	2.52	0.15
		2	16.01	0.11	1.48	1.11	16.43	0.06
		3	14.38	1.32	1.51	16.15	1.29	0.16
		4	17.54	0.00	0.06	13.36	4.21	0.03
	5	1	5.22	12.04	0.34	3.44	13.07	1.09
		2	13.54	3.41	0.25	7.54	7.28	2.38
		3	9.50	5.05	3.05	7.32	9.27	1.01
		4	12.01	5.55	0.04	11.32	6.17	0.11
	6	1	0.39	12.24	4.57	9.18	5.15	3.27
		2	0.03	17.17	0.40	14.15	2.11	1.34
		3	0.00	10.11	7.49	14.59	15.53	0.54

		4	0.15	17.45	0.00	16.11	1.38	0.11
	7	1	10.39	6.49	0.32	14.55	0.49	2.16
		2	6.26	10.45	0.49	16.50	0.12	0.58
		3	0.00	17.53	0.07	17.06	0.29	0.25
		4	7.01	10.25	0.34	17.25	0.18	0.17
	8	1	13.09	1.59	2.52	16.41	1.16	0.03
		2	11.02	5.15	1.43	16.43	0.05	1.12
		3	11.59	5.36	0.25	17.47	0.00	0.13
		4	15.58	1.26	0.36	16.56	1.04	0.00
	9	1	10.31	5.47	1.42			
		2	10.33	5.04	2.23			
		3	8.18	6.00	3.42			
		4	13.17	0.36	0.19			
	10	1	7.49	9.55	0.16			
		2	6.35	11.11	0.14			
		3	11.16	5.11	1.33			
		4	6.03	11.50	0.07			
8 mg/L	1	1	15.23	2.00	0.37	14.46	2.53	0.22
		2	7.58	10.02	0.00	4.41	13.19	0.00
		3	12.31	5.29	0.00	13.55	3.52	0.14
		4	12.39	5.18	0.03	15.59	1.54	0.08
	2	1	14.49	3.03	0.08	16.06	1.22	0.32
		2	10.55	1.56	5.09	17.44	0.58	0.14
		3	16.45	0.59	0.16	17.41	0.00	0.19
		4	15.50	1.16	0.54	17.30	0.09	0.21
	3	1	17.20	0.30	0.10	14.49	2.31	0.40
		2	16.18	0.08	1.34	16.17	1.10	0.33

		3	17.29	0.06	0.25	14.26	2.57	0.37
		4	17.34	0.06	0.20	16.45	0.34	0.41
	4	1	17.30	0.17	0.13	0.42	17.18	0.00
		2	16.23	1.33	0.31	0.55	17.05	0.00
		3	17.41	0.10	0.09	0.31	17.29	0.00
		4	16.48	0.29	0.44	3.02	14.58	0.00
	5	1	5.05	12.36	0.19	4.08	13.05	0.47
		2	12.36	5.19	0.05	5.27	7.03	1.30
		3	9.15	8.27	0.18	2.19	15.38	0.03
		4	5.30	11.39	0.51	6.57	9.27	1.36
	6	1	5.50	7.05	5.05	7.55	8.09	1.56
		2	0.00	18.00	0.00	9.52	6.59	1.09
		3	5.09	12.23	0.28	10.39	4.36	2.45
		4	1.10	16.35	0.15	14.54	1.52	1.14
	7	1	3.11	9.27	5.22	10.55	6.15	0.50
		2	5.30	9.22	3.08	11.38	4.32	0.50
		3	10.21	0.00	1.01	15.30	1.56	0.34
		4	2.57	13.10	1.53	16.00	1.38	0.22
	8	1	15.31	1.09	1.20	10.31	7.22	0.07
		2	13.33	1.57	2.30	13.45	3.48	0.28
		3	16.42	0.27	0.51	14.31	3.20	0.09
		4	15.36	0.28	1.56	16.39	1.12	0.09
	9	1	15.37	1.36	0.47			
		2	13.00	1.08	3.52			
		3	16.57	1.45	0.18			
		4	17.11	0.31	0.18			
	10	1	12.18	4.58	0.44			

		2	9.56	7.17	0.47	
		3	9.46	7.36	0.18	
		4	11.10	6.42	0.08	

The swimming activity was recorded during 10’ for each group of aquaria (TAN treatment) using a Sony HD (HDR-SR1E) camera. The experiment lasted for eight days, and recordings were made on alternative days (four days) between 9:00 and 12:00 AM.
