# Supplementary material for: Improving water quality does not guarantee fish health: Effects of ammonia pollution on the behaviour of wild-caught pre-exposed fish
Source: PLoS One. 2021 Aug 9;16(8):e0243404. doi: 10.1371/journal.pone.0243404 (PMC8351958; doi:10.1371/journal.pone.0243404)
Supplement: S3 Table — (PDF) [file pone.0243404.s003.pdf]

S3 Table. Data collected from the feeding behaviour records of each specimen.

			Pre-exposed fish			Non pre-exposed fish		
**TAN treatment**	**Specimen**	**Recording day**	**Latency (s)**	**Voracity**	**Satiety**	**Latency (s)**	**Voracity**	**Satiety**
0 mg/L	1	1	24.80	3	7	8.11	7	10
		2	21.06	2	4	9.4	10	12
		3	23.61	4	5	2.51	13	14
		4	14.92	3	3	3.05	12	13
	2	1	7.56	2	5	8.21	6	8
		2	28.95	4	8	263.05	2	2
		3	47.96	3	11	71.96	4	4
		4	3.55	4	5	4.84	3	3
	3	1	8.95	3	4	15.78	4	4
		2	29.68	7	8	9.67	4	4
		3	55.28	4	5	2.73	6	7
		4	3.30	6	10	17.52	4	4
	4	1	20.78	2	2	17.25	6	6
		2	17.15	2	3	9.34	11	11
		3	8.38	3	4	6.54	7	7
		4	3.52	4	5	9.62	6	7
	5	1	11.96	4	4	22.74	7	8
		2	4.86	4	5	3.11	7	8
		3	5.06	5	9	4.19	5	6
		4	3.08	4	5	13.73	5	6
	6	1	6.91	2	4	41.45	3	3
		2	61.61	3	4	6.51	4	4
		3	21.11	4	5	3.15	6	6

		4	41.72	3	3	6.52	8	8
	7	1	32.88	3	4	8.6	5	5
		2	60.08	2	3	4.56	4	4
		3	16.05	3	3	1.26	6	7
		4	32.33	2	3	5.5	5	5
	8	1	6.11	2	2	4.47	2	2
		2	22.94	2	2	3.51	6	6
		3	42.46	4	5	6.39	5	5
		4	9.13	2	3	7.34	3	3
	9	1	3.38	7	11			
		2	2.03	7	10			
		3	6.21	9	12			
		4	1.93	8	16			
	10	1	3.00	7	11			
		2	2.61	6	8			
		3	4.90	4	6			
		4	8.33	8	10			
1 mg/L	1	1	54.87	0	0	6.01	8	8
		2	28.53	1	1	8.98	8	9
		3	7.34	5	6	1.38	6	7
		4	10.49	4	5	13.52	6	6
	2	1	36.06	0	0	17.24	1	1
		2	145.17	0	0	88.49	0	0
		3	99.00	2	4	18.22	2	2
		4	87.33	2	2	33.49	0	0
	3	1	9.73	0	0	6.84	8	8
		2	24.56	0	0	3.67	4	4

		3	5.70	1	1	2.79	7	7
		4	4.54	1	1	2.87	5	5
	4	1	15.61	4	4	17.62	2	2
		2	44.70	12	12	1.29	2	2
		3	9.71	5	6	4.75	2	2
		4	49.45	6	6	2.14	6	6
	5	1	43.95	1	1	10.24	7	7
		2	9.83	3	4	1.75	1	1
		3	5.54	3	4	3.27	5	5
		4	5.11	2	2	2.78	7	7
	6	1	11.01	1	1	15.91	6	6
		2	96.50	1	1	7.59	4	4
		3	6.45	1	1	5.49	4	4
		4	3.19	2	2	2.88	5	5
	7	1	9.76	1	1	19.29	3	3
		2	71.43	0	0	55.8	5	5
		3	24.19	0	0	12.88	4	4
		4	2.84	1	1	2.75	2	2
	8	1	9.04	0	0	52.03	3	3
		2	4.65	1	1	37.65	2	2
		3	7.70	3	4	56.79	3	3
		4	2.55	1	1	8.75	5	5
	9	1	21.83	0	0			
		2	142.29	0	0			
		3	9.06	0	0			
		4	18.39	0	0			
	10	1	39.21	2	2			

		2	7.93	2	2			
		3	8.88	3	4			
		4	3.90	2	2			
5 mg/L	1	1	57.90	2	2	3.84	10	12
		2	259.79	0	0	53.04	10	11
		3	124.82	2	2	8.93	10	10
		4	9.91	3	4	42.97	11	11
	2	1	21.65	0	0	2.45	12	13
		2	69.81	2	2	3.9	8	8
		3	13.13	2	2	2.48	5	5
		4	12.21	2	2	2.8	5	5
	3	1	64.13	0	0	3.6	7	7
		2	131.22	0	0	24.87	4	4
		3	120.42	1	1	1.98	4	4
		4	44.80	1	1	0.76	3	3
	4	1	3.50	2	2	17.44	5	5
		2	1.95	2	2	41.06	3	3
		3	3.03	3	4	12.71	4	4
		4	18.75	3	4	1.7	5	5
	5	1	25.35	0	0	19.34	4	4
		2	11.73	4	5	6.34	3	3
		3	2.09	2	2	3.48	5	5
		4	9.60	2	2	1.26	2	2
	6	1	126.13	0	0	10.1	6	6
		2		0	0	22.16	4	4
		3		0	0	5.83	2	2
		4	70.29	5	5	8.91	8	8

	7	1	37.70	0	0	8.48	6	6
		2	352.91	0	0	5.33	5	5
		3	83.16	1	1	1.23	8	10
		4	34.46	2	2	1.06	7	7
	8	1	49.02	0	0	7.97	3	3
		2	83.72	0	0	379.77	0	0
		3	83.32	1	1	0.86	1	1
		4	15.29	2	2	0.78	2	2
	9	1	12.78	3	3			
		2	10.48	1	2			
		3	20.70	2	2			
		4	27.99	2	2			
	10	1	14.90	2	2			
		2	16.03	1	1			
		3	15.83	4	5			
		4	2.60	3	4			
8 mg/L	1	1	5.62	4	4	27.61	3	3
		2	6.43	1	1	61.83	4	4
		3	8.10	0	0	13.95	4	4
		4	11.45	1	1	3.33	3	4
	2	1	11.52	2	2	7.71	5	5
		2	7.52	0	0	3.7	4	5
		3	7.33	0	0	4.69	4	4
		4	3.05	1	3	9.21	4	4
	3	1	5.13	1	1	12.99	5	5
		2	32.14	1	1	9.55	4	4
		3	9.93	0	0	4.26	6	6

		4	5.31	0	0	7.25	4	4
	4	1	11.28	1	1	284.74	1	1
		2	9.21	0	0	41.51	1	1
		3	6.25	0	0	245.29	0	0
		4	7.25	3	5	97.55	1	1
	5	1	58.30	1	1	17.08	3	3
		2	99.61	0	0	21.39	1	1
		3	18.23	0	0	22.13	1	1
		4	37.58	1	1	7.5	6	6
	6	1	37.00	1	1	14.51	4	4
		2	351.45	0	0	4.72	4	4
		3	3.35	1	1	4.74	1	1
		4	30.78	0	0	2.5	4	4
	7	1	31.86	1	1	2.74	2	2
		2	453.89	0	0	1.26	2	2
		3	94.28	1	1	5.63	2	2
		4	148.08	0	0	3.63	4	4
	8	1	33.26	1	1	15.09	2	2
		2	117.25	0	0	3.61	2	2
		3	17.81	0	0	30.34	3	3
		4	23.47	1	1	4.34	3	3
	9	1	34.29	2	3			
		2	86.76	5	7			
		3	10.86	6	7			
		4	8.71	11	12			
	10	1		0	0			
		2	312.09	0	0			

		3	386.64	0	0	
		4	25.21	0	0	

The feeding behaviour was recorded (until fish stopped eating) for each group of aquaria (TAN treatment) using a Sony HD (HDR-SR1E) camera. The experiment lasted for eight days, and recordings were made on alternative days (four days) between 9:00 and 12:00 AM.
