# Supplementary material for: Improving water quality does not guarantee fish health: Effects of ammonia pollution on the behaviour of wild-caught pre-exposed fish
Source: PLoS One. 2021 Aug 9;16(8):e0243404. doi: 10.1371/journal.pone.0243404 (PMC8351958; doi:10.1371/journal.pone.0243404)
Supplement: S4 Table — (PDF) [file pone.0243404.s004.pdf]

S4 Table. Data collected from the biochemical determination.

	Pre-exposed fish					Non pre-exposed fish				
TAN Treatment	LPO	GSH	CAT	LDH	GST	LPO	GSH	CAT	LDH	GST
0 mg/L						1.39	24.59	0.76	1.69	342.22
	1.78		0.53	1.56	342.57			0.54	2.02	438.19
			0.45	2.27	375.27			0.67	2.43	387.48
	1.16	15.36	0.46	1.95	355.91		23.71	0.74	2.21	357.80
								1.08	1.73	378.47
	1.59	16.31	0.49	2.21	463.77	1.56	24.12	0.91	2.27	324.34
		18.88	0.51	1.84	396.72		21.68	0.77	1.74	310.92
						1.08				
	1.41	19.24	0.30	1.88	462.75					
	0.73		0.40	2.09	450.55					
1 mg/L	1.59	20.57	0.35	1.80	443.41	0.89				
	1.39	18.83	0.48	2.16	446.06		19.37	1.25	3.16	518.03
							31.15	0.74	2.28	519.88
	1.68	21.30	0.31		506.53		24.50	0.80	2.05	429.28
				1.03	309.69		30.36	0.65	2.09	408.65
								0.82	2.41	469.75
			0.29	1.94	460.59	0.92				
			0.38	2.03	489.04	1.70				
	1.07	23.91	0.37	1.70	473.80					
5 mg/L		16.49	0.38	2.03	436.21	1.72		0.47	1.44	348.82
		20.59	0.51	2.16	443.30	1.41	23.58	0.75	1.66	314.99

						1.50	28.11	0.62	2.09	445.57
	1.37	18.31	0.83	2.41	416.74	1.14	27.12	0.87	2.43	357.10
			0.59	1.68	395.35	1.47	28.31	0.67	1.47	391.19
	2.06		0.41	1.61	457.36		25.91	0.84	2.10	404.53
			0.58	2.17	485.04		28.14	0.91	1.87	356.45
	1.88		0.59	2.19	363.80	1.15		0.81	2.18	452.24
			0.92	1.77	368.59					
	1.74		0.70	2.04	464.66					
8 mg/L			0.70	2.33	478.28	1.33	24.83	0.78	2.25	371.88
			0.80	2.43	453.45	1.18	24.14	0.97	1.73	377.12
	1.57		0.70	2.11	481.05	1.14	28.10	0.69	1.72	367.99
	2.53	23.23	0.53	2.28	498.96	1.44		0.54	2.30	505.31
	3.08	17.12	0.47	1.91	334.18		27.35	1.06	1.63	392.92
	3.68	21.30	0.53	1.43	452.77			0.90	2.24	374.84
							32.47	0.64	1.74	436.02
			0.43	1.45	403.50		24.79	1.16	2.46	500.58
	4.07	17.75	0.51	1.86	501.82					
	3.96	21.97	0.64	1.86	379.30					

Biomarkers were analysed in the liver tissue for each individual fish.
